# Nucleus accumbens functional connectivity changes underlying alcohol expectancies in bipolar disorder and prospective alcohol outcomes: a within-subject randomized placebo-controlled alcohol administration fMRI study

**DOI:** 10.3389/fnins.2025.1549295

**Published:** 2025-04-09

**Authors:** Elizabeth T. C. Lippard, Dylan E. Kirsch, Vanessa Le, Skyler Lee, Nadia Bibb, Kaitlyn Meek, Raquel Kosted, Ansley Huffman, J. R. C. Almeida, Kim Fromme, Stephen M. Strakowski

**Affiliations:** ^1^Department of Psychiatry and Behavioral Sciences, University of Texas, Austin, TX, United States; ^2^Department of Psychology, University of Texas, Austin, TX, United States; ^3^Waggoner Center for Alcohol and Addiction Research, University of Texas, Austin, TX, United States; ^4^Interdisciplinary Neuroscience Program, University of Texas, Austin, TX, United States; ^5^Institute of Early Life Adversity Research, University of Texas, Austin, TX, United States; ^6^Department of Psychology, University of California, Los Angeles, Los Angeles, CA, United States; ^7^Department of Psychiatry, University of Texas Southwestern Medical School, Dallas, TX, United States; ^8^Department of Psychiatry, Indiana University School of Medicine, Indianpolis, IN, United States

**Keywords:** bipolar disorder, placebo, alcohol expectancies, fMRI, drinking, prospective

## Abstract

**Introduction:**

Alcohol use disorder (AUD) occurs at higher rates in individuals with bipolar disorder compared to the general population. A paucity of data are available on specific mechanisms that may contribute to bipolar and AUD co-occurrence. We recently reported differences in alcohol expectancies and placebo response during alcohol administration in early-stage bipolar disorder, compared to healthy young adults. This current report investigated subjective and neural response following placebo beverage consumption in young adults with bipolar disorder.

**Methods:**

As part of a within-subject placebo-controlled alcohol administration study, 54 young adults (53% with bipolar disorder type I, age_*mean*_ + SD = 23 + 2 years, 64% female) completed resting state functional MRI (rsfMRI) scans at baseline (pre-beverage) and following placebo and alcohol consumption (counter-balanced). Participants completed subjective response measures during placebo and alcohol beverage conditions. Between-group differences in subjective response and placebo-related changes in functional connectivity of the Nucleus Accumbens (NAc) with other brain regions, compared to a pre-beverage rsfMRI baseline condition, were investigated. Fisher-transformed correlation coefficients between ROIs and seed-to-clusters showing a significant group-by-condition (placebo, pre-beverage rsfMRI) interaction were calculated. Associations with prospective alcohol use and problems were explored in a subgroup with longitudinal data.

**Results:**

Young adults with bipolar disorder reported greater intoxication during the placebo condition, compared to healthy young adults (main effects of group: *p* < 0.05). Compared to pre-beverage rsfMRI, the placebo condition related to increased connectivity between bilateral NAc and regions within the sensorimotor network in bipolar disorder. Comparison participants showed the opposite pattern of placebo-related changes in connectivity (group-by-condition, p-FDR < 0.05). Greater anxiolytic effects endorsed during placebo and associated increases in NAc functional connectivity related to greater alcohol use and alcohol problems at follow-up in bipolar disorder (*p* < 0.05).

**Discussion:**

Results suggest differences in placebo response in bipolar disorder, including distinct neural correlates, that may relate to prospective alcohol use/problems. Given the theoretical association between placebo response and self-reported alcohol expectancies, findings could open the door to interventions aimed at changing expectancies.

## 1 Introduction

A diagnosis of bipolar disorder coincides with one of the highest prevalence rates of alcohol use disorder (AUD) of any psychiatric diagnosis (Conway et al., 2006). While a 20%–40% prevalence of AUD in bipolar disorder is suggested (Di Florio et al., 2014; Hunt et al., 2016; Nery et al., 2014; Simhandl et al., 2016), estimates as high as 60% prevalence of lifetime AUD in bipolar disorder have been reported (Regier et al., 1990). This co-occurrence is associated with a more pernicious illness course than when either condition is presented on their own, ranging from worse mood episodes to greater cognitive deficits and a higher risk of suicide (Cardoso et al., 2016; Finseth et al., 2012; Goldberg, 2001; Goldstein et al., 2006; McGrady et al., 2017; Messer et al., 2017; Nery et al., 2014; Oquendo et al., 2010; Sanchez-Moreno et al., 2009; Strakowski et al., 2005; Strakowski et al., 2000). Despite the widely recognized association between these conditions and associated clinical sequelae, few developmental studies exist investigating behavioral and neural mechanisms that may underlie risk and serve as early intervention targets. However, emerging work suggests youth with bipolar disorder may show differences in sensitivity to alcohol that may contribute to alcohol use and risk for development of AUD (Kirsch et al., 2023a; Lippard et al., 2023; Tretyak et al., 2021; Yip et al., 2012).

Variability in how individuals respond to alcohol is suggested to increase risk for developing AUD (King et al., 2021; King et al., 2011; King et al., 2014; Quinn and Fromme, 2011; Ray et al., 2010)—however, prior studies in this area typically excluded individuals with bipolar disorder. We recently completed a within-subject placebo-controlled alcohol administration study in young adults with bipolar disorder and healthy comparison young adults. Preliminary data suggested increased sensitivity to alcohol in young adults with bipolar disorder, compared to healthy comparison young adults (Lippard et al., 2023), with greater positive stimulating effects of alcohol and greater past month alcohol use associated with changes in nucleus accumbens (NAc) functional connectivity during the alcohol compared to placebo condition (Kirsch et al., 2023a). Moreover, the most robust differences observed in subjective response to alcohol were main effects of group, with young adults with bipolar disorder reporting greater subjective experience of intoxication during both the placebo and alcohol beverage conditions (Lippard et al., 2023), suggesting variability in alcohol expectations in bipolar disorder.

Placebo response is driven in part by alcohol expectancies, i.e., a person’s belief about what happens when they consume alcohol. Alcohol expectancies correlate with alcohol use in healthy populations (Fromme et al., 1993; Hasking et al., 2011; Kilbey et al., 1998) and recent work suggested greater neural response to a placebo beverage condition predicts greater alcohol problems in healthy young adults over a 5 years period (McKenna et al., 2022). However, this prior study did not include a non-placebo fMRI condition to distinguish if neural responses are specific to placebo manipulation or a baseline trait. We previously reported NAc functional connectivity changes during resting state fMRI (rsfMRI) that were associated with subjective level of intoxication during placebo beverage consumption, compared to a pre-beverage rsfMRI scan, in healthy young adults (Kirsch et al., 2023b), suggesting striatal reward systems may underlie alcohol expectancies. This extends prior work suggesting a neural basis for alcohol expectancies, i.e., alcohol expectancies positively relate to resting limbic and striatal functional connectivity (Le et al., 2022; Le et al., 2020; Zhornitsky et al., 2018), to suggest placebo-related change in striatal connectivity can be distinguished from trait neural phenotypes that may indirectly relate to alcohol expectancies as previously discussed (Kirsch et al., 2023b). It is unknown whether similar placebo-related changes in NAc functional connectivity are observed in bipolar disorder and associated with elevated placebo response.

This study is a secondary data analysis from a placebo-controlled alcohol administration study in bipolar disorder (NCT04063384). As part of the study design, a rsfMRI scan was collected prior to and following both alcohol and placebo administration. This study is hypothesis driven. We hypothesized that young adults with bipolar disorder, compared with healthy comparison young adults, would report greater subjective effects of intoxication during both alcohol and placebo conditions with increased perceived intoxication during placebo, compared to a pre-beverage fMRI scan, relating to greater increases in NAc functional connectivity with ventral prefrontal regions of interest (ROIs) consistent with prior work in healthy young adults (Kirsch et al., 2023b). Additionally, we conducted an exploratory analysis of placebo-associated changes in NAc functional connectivity that may extend outside of *a priori* ROIs. By focusing on the placebo manipulation, we aimed to investigate the neural underpinnings of expectancy-related effects that occur following alcohol consumption (in the absence of alcohol pharmacokinetics). This study extends our prior work to investigate neural underpinnings of alcohol expectancies in young adults with bipolar disorder. As it is unknown if placebo response—and underlying neural systems—relates to prospective alcohol use/problems in bipolar disorder, a subset of individuals provided longitudinal data on alcohol outcomes and associations between placebo response and alcohol-related outcomes were also explored. We hypothesized greater sensitivity to placebo manipulation—and associated changes in NAc functional connectivity—in bipolar disorder would relate to greater alcohol use/problems over time. A better understanding of factors that contribute to alcohol misuse and problems over time could support novel interventions for individuals with bipolar disorder (e.g., alcohol expectancy challenges or neuromodulation targets).

## 2 Materials and methods

### 2.1 Participants

Participants were recruited from Central Texas between July 2019 and February 2024 as previously described (Lippard et al., 2023). Participants completed a fMRI session prior to beverage sessions and another fMRI session following placebo and alcohol beverage consumption. Sixty participants enrolled into the parent study (50% with bipolar disorder type I). A power analysis prior to the start of study suggested 30 individuals per group would provide adequate power for testing our hypothesis. Specifically, at an alpha = 0.05, 30 subjects per group will provide > 80% statistical power to detect a within-subject ES d = 0.5 and a between group ES d = 0.7. Five participants were excluded from this secondary analysis (two with bipolar disorder and three healthy comparison young adults) owing to placebo manipulation issues. Additionally, one healthy comparison subject was excluded because they later revealed a history of a major depressive episode. The final dataset for this secondary analysis therefore included 28 young adults with bipolar I disorder and 26 healthy comparison young adults. We also excluded one additional healthy comparison young adult from the rsfMRI analysis as the image acquisition protocol changed after the first enrolled participant’s MRI scan. Our final resting state fMRI analysis included 28 young adults with bipolar disorder and 25 healthy comparison young adults. Participants with bipolar disorder were euthymic and stable on medication at the time of fMRI and beverage sessions. Individuals in the healthy comparison subgroup had no history of psychiatric hospitalizations; neurodevelopmental, affective, or psychotic disorder; or > 1 month of lifetime psychotropic medication. Exclusion criteria for all participants included: full scale intelligence quotient (IQ) < 85; contraindication to MRI scanning; significant head trauma (loss of consciousness for greater than 5 min); positive pregnancy test; history of severe AUD; a current SUD (other than alcohol, cannabis, or nicotine). Additional exclusion criteria (all participants) included heart trouble; high blood pressure; history of heart attack; diabetes; liver disease; ever being in an abstinence-oriented treatment program for alcohol use; reporting wanting to quit drinking but not being able to; any medical, religious, or other reasons for not drinking alcohol; an adverse reaction to alcoholic beverages (e.g., severe flushing; seizure); an Alcohol Use Disorder Identification Test (AUDIT) score > 15 (assessed at time of phone screen), and unwillingness to have friend/family member drive them home after beverage sessions. All participants had to report consuming four (men) or three (women) or more drinks on a drinking occasion in the past year. Inclusion/exclusion criteria were defined to minimize risk associated with an alcohol administration study in accordance with the NIAAA Guidance for Conducting Alcohol Administration Studies with Human Participants. Specifically, individuals needed to drink to the point of intoxication they would be dosed to in the lab (0.08g%) to minimize individuals having a negative reaction to alcohol, but not drink too much or endorse alcohol-related problems where it would be an ethical concern to provide alcohol in the lab (e.g., someone reporting wanting to quit drinking but not being able to). Study procedures were approved by the Institutional Review Board at the University of Texas at Austin.

### 2.2 Clinical and recent alcohol assessment

The Structured Clinical Interview for DSM 5, Research Version (SCID-5-RV) was used to confirm presence of a prior manic episode in individuals with bipolar disorder. The Wechsler Abbreviated Scale of Intelligence matrix reasoning and vocabulary subtests were used as a measure of IQ. The Timeline Follow-Back [TLFB (Sobell and Sobell, 1992)] was used to measure recent alcohol, tobacco, and marijuana use over the past 30 days. Total drinks, number of days drinking, and average number of drinks per drinking day was calculated. We also calculated maximum number of drinks consumed on a drinking day and number of heavy episodic drinking episodes (> 5 drinks for men and > 4 drinks for women). Participants completed the Barratt Impulsiveness Scale-11 [BIS-11 (Patton et al., 1995)].

### 2.3 Alcohol and placebo beverage administration procedures

Following enrollment and the pre-beverage fMRI scan, participants completed an alcohol and placebo beverage condition on two separate days (counter-balanced). Beverage consumption occurred in a private room in the Biomedical Imaging Center with research staff present, as previously described (Lippard et al., 2023). The order of beverage session was randomized. Participants were informed they would be drinking alcohol on both beverage days and could receive different amounts of alcohol on each respective day, but they would not be dosed to exceed a breath alcohol concentration (BrAC) of 0.08 g%. Participants were blinded to the beverage condition they would receive on each respective day, as described in previous studies (Quinn and Fromme, 2016). The Beck Depression and Anxiety Inventories (BDI and BAI, respectively) were completed as soon as individuals arrived on each drinking day. Participants were asked to fast from food for 4 h prior to drinking and then consumed a weight-adjusted snack of pretzels prior to beverage consumption. This approach delays the rate of alcohol absorption, increasing the duration of the ascending limb of the blood alcohol concentration curve (Jones and Jonsson, 1994). On the alcohol condition day, participants were dosed to a 0.08 g% target BrAC based on the participants’ age, sex, height, and weight (Cofresí et al., 2020; Corbin et al., 2021). Participants were given 20 min to consume two beverages (1:3 mixture of 80 proof vodka to mixer, 10 min per beverage). The mixer contained cranberry juice, diet seven-up, and lime juice. After 20 min of drinking and a 10 min absorption period (during which participants rinsed their mouth with alcohol free mouth wash), BrAC, heart rate, and measures of subjective response to alcohol was collected. The placebo and alcohol conditions were identical per standardized protocols (Cofresí et al., 2020; Corbin et al., 2021; Quinn and Fromme, 2016) with decarbonated tonic water stored in Absolute vodka bottles used in place of vodka for the placebo condition. For both beverage sessions, participants remained seated during the beverage session but saw vodka (or tonic) being poured out of an Absolute bottle (visual cue), the table was wiped down with tequila before the participant entered the room (olfactory cue), and an alcohol floater was added to beverages (gustatory cues). There were minimal environmental (i.e., alcohol-related) cues during the beverage session with the exception of participants seeing their drinks being made. Post-beverage BrAC assessment during the placebo condition was yoked to timing of BrAC collection during the alcohol session.

Subjective response to alcohol (or placebo) was collected on each beverage day with the Subjective Effects of Alcohol Scale [SEAS (Morean et al., 2013a)]. Four SEAS continuous subscale scores were calculated: positive valence/positive arousal (“lively, talkative, fun, funny”); positive valence/negative arousal (“mellow, relaxed, secure, calm”); negative valence/positive arousal (“aggressive, rude, demanding”); and negative valence/negative arousal (“woozy, dizzy, wobbly”). BrAC, heart rate, and the SEAS was collected before each beverage session began (BrAC was 0.0 g% for all participants prior to beverage consumption) and after beverage consumption/absorption [immediately prior to the fMRI scan (defined as pre-scan BrAC, heart rate, and subjective response)]. BrAC, heart rate, and subjective response were collected again immediately after participants exited the scanner (defined as post-scan BrAC, heart rate, and subjective response). Changes in SEAS at pre-scan and post-scan BrAC, compared to pre-beverage baseline SEAS scores on each respective day, was calculated for both beverage conditions. We collected a modified version of the Drug Effects Questionnaire [DEQ (Morean et al., 2013b)] at pre- and post-scan BrAC which assessed the extent to which participants (1) felt drunk, (2) felt effects of alcohol, (3) liked how they were feeling, and (4) wanted more of what they had consumed. At pre-scan BrAC, we asked participants to estimate the number of standard alcohol drinks they were served during the experiment which served as a placebo manipulation check. This study excluded anyone that did not report having consumed at least one alcohol beverage during their placebo manipulation. Following post-scan BrAC data collection, BrAC readings were continued every 30 min until participants were below a 0.04 g% BrAC during the alcohol condition.

### 2.4 MRI acquisition and preprocessing

All imaging occurred with a 3-Tesla Siemens VIDA MR scanner (Siemens, Erlangen, Germany) using a 64-channel head coil at the University of Texas at Austin Biomedical Imaging Center as previously described (Kirsch et al., 2023b). Briefly, a sagittal three-dimensional MPRAGE T1-weighted sequence was acquired with parameters: repetition time (TR) = 2,400 ms, echo time (TE) = 2.18 ms, matrix = 208 × 300 × 320, flip angle = 8°; field of view = 167 mm × 240 mm × 256 mm, 0.8 mm slices and one average with isotropic voxel geometry (0.8 mm × 0.8 mm × 0.8 mm). A rsfMRI scan was collected prior to beverage consumption and following both alcohol and placebo beverage conditions. For this analysis, we only examined pre-beverage and post-placebo rsfMRI data to focus on group differences in placebo response. FMRI data were collected with a single-shot echo-planar imaging sequence aligned with the anterior-posterior commissure plane with multiband factor: 6, TR = 778 ms, TE = 30 ms, matrix = 86 × 86, flip angle = 52°, field of view = 215 mm × 215 mm, and 60 2.5 mm thick slices without gap (voxel size = 2.5 mm × 2.5 mm × 2.5 mm). During the rsfMRI scan, participants viewed the Headspace Studios’ Inscapes video (6 min). The video contains an inanimate object changing shape and has been used previously in rsfMRI studies, including those involving clinical populations (Chung et al., 2023; Plewnia et al., 2021). While some differences in viewing the Inscapes video, compared to traditional rsfMRI paradigms, are reported (Vanderwal et al., 2015), we utilized the video to minimize motion, improve signal to noise (Vanderwal et al., 2017; Vandewouw et al., 2021), and reduce concerns that alcohol or placebo beverage consumption may alter an individual’s thoughts enough, which could limit our ability to interpret differences across conditions.

The CONN toolbox^1^ (Whitfield-Gabrieli and Nieto-Castanon, 2012) was used for rsfMRI data preprocessing as previously described (Kirsch et al., 2023b). Denoising included aCompCor (anatomical component analysis correction) regression (Behzadi et al., 2007) followed by quadratic detrending and band-pass filtering (0.008–1 Hz). Cerebrospinal fluid, white matter, six motion parameters and their first derivatives, scrubbing parameters, and condition effects were included as nuisance regressors. We utilized both ROI-to-ROI and seed-to-voxel based approaches to investigate group differences in NAc changes underlying placebo response (further detailed below).

## 2.5 Statistical analysis

### 2.5.1 Demographic and clinical characteristics and between/within group differences during beverage conditions

Between group differences in continuous variables were assessed with *t*-tests and Mann-Whitney-Wilcoxon tests, as appropriate. Categorical variables were assessed with Chi-square or Fisher’s exact tests, as appropriate. Group differences and group-by-time interactions on BrAC during the alcohol condition were investigated with BrAC (pre- and post-scan) as repeated within-subject variables. Parallel models were used to investigate changes in heart rate, with heart rate on the alcohol condition day (baseline, pre-scan, and post-scan) as repeated within-subject variables. We repeated this model with heart rate collected on the placebo day to explore if the placebo manipulation related to changes in heart rate across the study session. Mixed models were used to investigate group-by-beverage condition interactions in time to BrAC collection and number of standard drinks estimated to have consumed at pre- and post-scan time points, as well as BAI and BDI total scores.

### 2.5.2 Between group differences in subjective response to alcohol

Group (bipolar, healthy)-by-condition (alcohol, placebo)-by-time of subjective response interactions on subjective response to alcohol were modeled, covarying beverage condition order, biological sex, and age as previously described (Lippard et al., 2023), with SEAS and DEQ subscale scores as the dependent variables. Time of subjective response (pre- and post-scan) and beverage condition (alcohol, placebo) were within-subject factors and group was an independent between-subject factor. Following no group-by-condition-by-time of subjective response interaction, the three-way interaction term was dropped and group-by-condition interactions were investigated. Following no two-way interaction, the two-way interaction term was dropped and main effects of group and condition were assessed. Findings for these planned analyses were considered significant at *p* < 0.05.

### 2.5.3 Between group differences in placebo response: ROI-ROI approach

A ROI-to-ROI bivariate correlation first-level analysis was performed using the CONN toolbox (Whitfield-Gabrieli and Nieto-Castanon, 2012) to calculate and extract fisher transformed correlation coefficients between *a priori* ROI-to-ROI pairs during the placebo and pre-beverage rsfMRI scans. ROIs were defined with the FSL Harvard Oxford Atlas and included ventromedial prefrontal cortex (vmPFC), subcallosal cingulate cortex (SCC), and bilateral NAc. We focused on these ROIs as previous data suggested placebo response in healthy young adults relate to increases in functional connectivity of the NAc with the vmPFC and SCC when viewing the Inscapes video (Kirsch et al., 2023b). We used mixed models to examine group-by-condition (placebo, pre-beverage rsfMRI)-by-NAc hemisphere (left, right) interactions on ROI-to-ROI functional connectivity (SCC and vmPFC modeled separately). RsfMRI condition (placebo, pre-beverage) and NAc hemisphere were within-subject factors, and ROI-to-ROI functional connectivity between the NAc with vmPFC and SCC were the dependent variables. Following no three-way interaction with hemisphere, the three-way interaction term was dropped, and group-by-condition interactions were investigated. Following no two-way interaction, the two-way interaction term was dropped and main effects of group and condition were investigated. Results of primary models were considered significant at *p* < 0.05. All models included biological sex, session order (if placebo came before or after the alcohol session), and age as covariates.

#### 2.5.4 Between group differences in placebo response: NAc seed region approach

The CONN toolbox was also used for a seed-to-voxel functional connectivity analysis of the NAc (pre-defined seed region) to investigate functional connectivity changes that may relate to placebo response outside of our *a priori* ROIs. The bilateral NAc seed was defined with the FSL Harvard Oxford Atlas as above. The mean time series for left and right NAc was used as a predictor in the multiple regression general linear model for each voxel during first-level modeling. Between group differences in placebo-related changes in NAc functional connectivity (placebo rsfMRI minus pre-beverage rsfMRI) were modeled at the second level. Cluster-extent based thresholding was used with results considered significant if they survived a primary threshold of voxel-wise *p* < 0.005 and a cluster-level extent threshold of *p* < 0.05 using the false discovery rate correction (p-FDR). Following a group difference, for the placebo and pre-beverage rsfMRI, fisher transformed correlation coefficients between NAc and cluster pairs were calculated and extracted. Data was extracted for both the left and right NAc seed region. We conducted a *post hoc* analysis with extracted data modeling group-by-condition-by-NAc hemisphere interactions. Age, biological sex, and order of the placebo condition (if it came before or after the alcohol condition) were included as covariates. If there were no interactions with hemisphere, the three-way interaction term was dropped, and group-by-condition interactions investigated. If a group-by-condition interaction was observed, the effects of condition (placebo, pre-beverage rsfMRI) were modeled (stratified by group). Main effects of group and condition were investigated if there was no two-way interaction. If a group-by-condition-by-hemisphere interaction was observed, the models were stratified by hemisphere, and group-by-condition interactions in each hemisphere were investigated as above.

#### 2.5.5 Sensitivity/exploratory analysis

*Post hoc* sensitivity analyses were conducted to confirm if group-by-condition interactions or main effects of group identified in above models remained significant after (1) covarying history of AUD (current or past) and (2) covarying history of cannabis use disorder (CUD; current or past). We also repeated analyses when controlling for anxiety symptoms (BAI total score) and when covarying total impulsivity (BIS total score). Anxiety symptoms and total BIS scores were included as covariates in *post hoc* sensitivity analyses since prior work suggests anxiety and impulsivity relates to variation in subjective response to alcohol (Chutuape and de Wit, 1995; Leeman et al., 2014) and greater anxiety and impulsivity is often reported in bipolar disorder (Lippard et al., 2023; Najt et al., 2007) and could represent a confound. Additionally, we completed a sensitivity analysis for models incorporating fMRI data above after removing one participant with bipolar disorder because of technical issues with the Inscapes video on their placebo beverage day.

We also explored effects of medication in individuals with bipolar disorder (any subclass of medication with more than five individuals on and off a respective medication subclass). Specifically, we investigated main effects of medication (on/off: antipsychotic, anticonvulsant, antidepressant/SSRI, or sedative/antihistamine) on subjective response variables that showed a between group difference in analyses above. Beverage condition and time of subjective response was considered repeated within-subject factors and medication-by-condition interactions were modeled covarying age, sex, and order of beverage session. If the interaction term was not significant, the two-way interaction term was dropped and main effects of a medication subclass investigated. We similarly investigated medication effects on placebo-associated change in ROI-to-ROI and seed-to-voxel functional connectivity that showed group differences above, with condition (pre-beverage and post-placebo consumption rsfMRI) as a repeated within-subject factor. Normality of data was investigated and for measures not normally distributed (e.g., SEAS positive valence/positive arousal), data was transformed and models repeated.

#### 2.5.6 Placebo-related changes in functional connectivity relations with subjective response to placebo

Placebo-adjusted changes in NAc seed-to-voxel based functional connectivity (placebo session minus pre-beverage rsfMRI session) were calculated for each participant for seed-to-cluster pairs that showed a significant group-by-condition interaction in the seed-to-voxel analysis above. Group-by-placebo-adjusted changes in functional connectivity interactions were modeled with subjective response subscales during placebo beverage condition (that showed a group difference in the subjective response analysis) as the dependent variables. Only pre-scan subjective response variables were used in this analysis to avoid effects of participants laying in the scanner and so results would be more comparable to other placebo-controlled studies. Age, sex, and order of placebo session were included as covariates. Following no significant group-by-placebo-adjusted change in NAc functional connectivity, the two-way interaction term was dropped and main effect of placebo-adjusted change in NAc functional connectivity was investigated. NAc hemisphere was considered a repeated within-subject variable unless interactions with NAc hemisphere were identified above. If group differences localized to a specific hemisphere, analysis was conducted only within the hemisphere showing group differences. Results were considered significant at *p* < 0.05.

#### 2.5.7 Exploratory analysis on prospective alcohol use/problems

A subset of participants (*n* = 20 with bipolar disorder and 16 healthy comparison young adults) to date have completed a follow-up clinical evaluation (on average 2 years after their baseline assessment/beverage sessions), which included reassessment of AUD symptoms on the SCID-5-RV and TLFB to assess past month alcohol use. Between group differences in number of AUD symptoms reaching a three on the SCID-5-RV and past month total drinking days, total drinks, maximum drinks on a drinking day, number of heavy episodic drinking days, and average drinks per drinking day (measured with the TLFB) over time were investigated. Time from baseline enrollment to clinical follow-up was included as a covariate with AUD symptoms at baseline and follow-up (or TLFB measures) considered repeated dependent variables. Additionally, we explored relations between subjective response to placebo (on any subjective response variable that differed by group) and alcohol use outcomes. Three healthy comparison participants were excluded from the exploratory analysis on subjective response to placebo since three of the healthy comparison individuals with longitudinal data did not believe the placebo manipulation. All individuals in the bipolar disorder subgroup with longitudinal data believed the placebo manipulation. Specifically, group-by-subjective response interactions were modeled with average drinks per drinking day at follow-up as the dependent variable, controlling for baseline average drinks per drinking day and time between baseline and follow-up assessment. Models were repeated for other TLFB variables. We also repeated these models to investigate relations between baseline subjective response during placebo with prospective symptoms of AUD on the SCID (controlling for baseline AUD symptoms), however, we only modeled this relation in the subgroup with bipolar disorder as there was a lack of variability in number of symptoms meeting threshold on the SCID at follow-up in the healthy comparison group. Parallel models described above were used to explore effects of placebo-related change in NAc functional connectivity (for any seed-to-cluster pair that showed a significant change in functional connectivity during the placebo session in bipolar disorder in above analyses). Functional connectivity of the left and right NAc was modeled separately. Results for these exploratory analyses were considered significant at *p* < 0.05.

## 3 Results

### 3.1 Demographic and clinical characteristics and between/within group differences during beverage conditions

Young adults with bipolar I disorder had greater depression and anxiety symptoms at enrollment, and higher impulsivity than healthy comparison young adults (*p* < 0.001; Table 1). No between group differences were observed in BrAC or group-by-time interactions on BrAC during the alcohol condition. No between group differences (or group-by-beverage condition interactions) were observed in time to pre-scan or post-scan BrAC collection. However, a main effect of beverage condition in time to post-scan BrAC collection (*p* = 0.02) was noted, with time to post-scan BrAC being longer during the alcohol, compared to placebo, condition day (94 min vs. 91 min, respectively). A main effect of time on heart rate was also observed during the alcohol condition (*p* = 0.01), with both groups showing an increase in heart rate over the course of the alcohol condition. No effect of time on heart rate was seen during the placebo session. Also, no group-by-time interactions in heart rate were noted. Young adults with bipolar disorder endorsed greater symptoms of depression and anxiety on their beverage days (main effect of group on BAI: *p* = 0.002, BDI: *p* < 0.001) compared to healthy young adults, but there were no differences in depression or anxiety symptoms between alcohol and placebo condition days. A main effect of condition was seen on number of standard drinks estimated to have consumed at pre-scan BrAC (*p* < 0.0001) but there was no group-by-condition interaction or main effect of group on number of standard drinks estimated to have consumed (placebo manipulation check).

**TABLE 1:** Between-group (bipolar disorder vs. healthy comparison young adults) differences in demographics, clinical mood symptoms, alcohol and substance use characteristics, and past month alcohol/cannabis/nicotine use were assessed using two-sample *t*-tests, Mann–Whitney-Wilcoxon tests, chi-square, or Fisher’s exact as appropriate.

		Healthy young adults (*N* = 26)	Bipolar disorder (*N* = 28)	*P*-value
**Demographics**				
	Mean age (SD)	22.6 (1.3)	23.4 (1.7)	0.06^L^
	Number of females (%)	15 (58)	20 (71)	0.4
	Mean WASI-II FSIQ^A^	120.7 (11.1)	116.1 (9.6)	0.1
	Hispanic (%)	6 (23)	12 (43)	0.1
	Non-Hispanic white (%)	10 (38)	11 (39)	
	Asian (%)	8 (31)	2 (7)	
	More than one race (%)	2 (8)	3 (11)	
**Clinical mood symptoms**				
	HDRS (SD)^B^	1.3 (1.5)	5.0 (3.4)	**< 0.0001^L^**
	HARS (SD)^C^	1.2 (1.5)	5.3 (4.6)	**< 0.0001^L^**
	YMRS (SD)^D^	0.3 (0.5)	1.0 (1.5)	0.07^K^
**Other clinical factors and comorbidities**				
	Lifetime suicide attempt (%)	–	12 (43)	N/A
	Lifetime anxiety disorders (%)^E^	–	14 (50)	N/A
	Total Barratt Impulsiveness Scale (SD)	57.7 (6.6)	73 (13.3)	**< 0.0001**
**Alcohol and substance use characteristics**				
	AUDIT (SD)^F^	4.8 (3.5)	5.7 (4)	0.4^L^
**Current alcohol use disorder**				
	Current alcohol use disorder, mild (%)	0	1 (4)	1.0^K^
**Past alcohol use disorder**				
	Past alcohol use disorder, mild (%)	1 (4)	3 (11)	0.6^K^
	Past alcohol use disorder, moderate (%)	0	2 (7)	0.5^K^
**Current substance use disorders**				
	Current cannabis use disorder, mild (%)	1 (4)	1 (4)	1.0^K^
	Current cannabis use disorder, severe (%)^G^	0	1 (4)	1.0^K^
**Past substance use disorders**				
	Past cannabis use disorder, mild (%)	0	3 (11)	0.2^K^
	Past cannabis use disorder, moderate (%)	0	3 (11)	0.2^K^
	Past cannabis use disorder, severe (%)	0	1 (4)	1.0^K^
	Past sedative use disorder, mild (%)	0	2 (7)	0.5^K^
	Past stimulant use disorder, severe (%)	0	1 (4)	1.0^K^
	Past hallucinogens use disorder, moderate (%)	0	1 (4)	1.0^K^
**Past month alcohol use^H^**				
	Total drinks (SD)	18.1 (21.5)	11.1 (8.3)	0.1
	Total drinking days (SD)	5.1 (4.5)	4.4 (2.6)	0.5
	Drinks/drinking days (SD)	3.2 (2)	2.5 (0.9)	0.1
**Past month cannabis Use^I^**				
	Cannabis user: Y/N (%)	6 (23)	13 (46)	0.07
	Cannabis use days (SD)^J^	10 (9.9)	10.7 (10.0)	0.9^L^
**Past month nicotine/tobacco use^I^**				
	Nicotine/tobacco user: Y/N (%)	2 (8)	4 (14)	0.7^K^
**Psychiatric medications**				
	Unmedicated at scan (%)	–	4 (14)	N/A
	Antipsychotic (%)	–	10 (36)	N/A
	Anticonvulsant (%)	–	13 (46)	N/A
	Antidepressant/SSRI (%)	–	9 (32)	N/A
	Stimulant (%)	–	8 (29)	N/A
	Lithium (%)	–	4 (14)	N/A
	Beta blocker (%)	–	3 (11)	N/A
	Sedative/antihistamine (%)	–	5 (18)	N/A

Between-group differences in comorbid anxiety disorders, lifetime suicide attempt, and psychotropic medications were not assessed as these were considered an exclusion criterion, and thus not present in the healthy comparison group. Bolded *p*-value statistics are values < 0.05 uncorrected.

^A^FSIQ-2 represents the composite score for the full-scale intelligence quotient comprising verbal comprehension and matrix reasoning subsets on the Wechsler Abbreviated Scale of Intelligence-Second Edition (WASI-II).

^B^Past week depression symptoms were measured using the Hamilton Depression Rating Scale (HDRS).

^C^Past week anxiety symptoms were measured using the Hamilton Anxiety Rating Scale (HARS).

^D^Past week mania symptoms were measured using the Young Mania Rating Scale (YMRS).

^E^Comorbid anxiety disorders included generalized anxiety disorder, social anxiety, social phobia, agoraphobia, and panic disorder.

^F^Alcohol Use Disorders Identification Test (AUDIT).

^G^One individual who met criteria for current severe cannabis use disorder (CUD; past year) had been in full remission for 6 months at time of rsfMRI and beverage sessions.

^H^Recent alcohol use was measured with the Timeline Follow Back Assessment (TLFB).

^I^Recent cannabis and tobacco use was measured with the Timeline Follow Back Assessment.

^J^Mean number of cannabis use days in individuals reporting past month cannabis use.

^K^Represents *p*-value calculated with Fisher’s exact test.

^L^Represents *p*-value calculated with a Mann–Whitney-Wilcoxon test.

### 3.2 Between group differences in subjective response to alcohol

**FIGURE 1:**
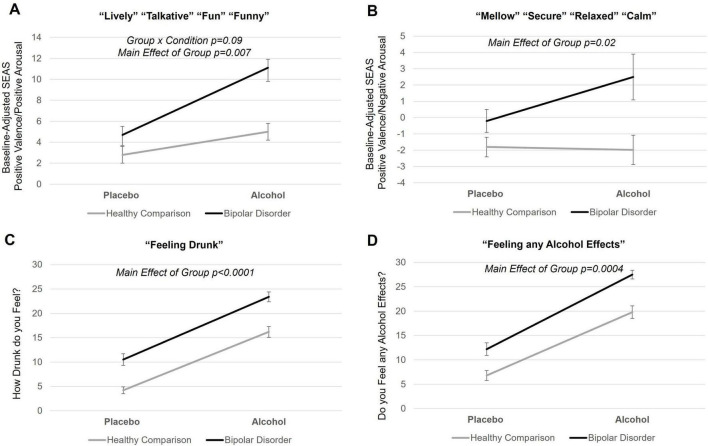
Subjective response to alcohol in young adults with bipolar disorder and healthy comparison young adults. **(A,B)** Mean self-report of subjective response to alcohol scores for young adults with bipolar disorder and healthy young adults during placebo and alcohol beverage conditions. Self-report of subjective response to alcohol measures included the Subjective Effects of Alcohol Scale (SEAS) and Drug Effects Questionnaire (DEQ). Each SEAS subscale score was baseline adjusted. **(C,D)** There was main effect of group on SEAS positive valence/positive arousal subscale (*p* = 0.007), SEAS positive valence/negative arousal subscale (*p* = 0.02), DEQ “feeling drunk” (*p* < 0.0001), and DEQ “feeling alcohol effects” (*p* = 0.0004), with young adults with bipolar disorder reporting greater scores across all these subjective response variables during both the alcohol and placebo condition. Means include pre- and post-scan subjective response as there was no time of subjective response interactions. Error bars represent standard errors. Black line indicates young adults with bipolar disorder; gray line indicates healthy comparison young adults.

A significant main effect of group was seen for SEAS positive valence/positive arousal [F = 7.6, *p* = 0.007, 95% confidence interval (CI) (1.1, 6.5)], SEAS positive valence/negative arousal [F = 6.1, *p* = 0.02, 95% CI (0.6, 5.5)], feeling drunk [F = 28.0, *p* < 0.0001, 95% CI (4.6, 10.1)], and feeling alcohol effects [F = 18.4, p0.00, 95% CI (3.6, 9.7)], with young adults with bipolar disorder feeling more effects across all subscale scores for both alcohol and placebo conditions (see Figure 1). Main effects of condition were observed for SEAS positive valence/positive arousal [F = 11.9, *p* = 0.0009, 95% CI (1.9, 7.0)], SEAS negative valence/negative arousal [F = 60.7, *p* < 0.0001, 95% CI (5.2, 8.7)], feeling drunk [F = 91.7, *p* < 0.0001, 95% CI (9.8, 15)], and feeling alcohol effects [F = 103.8, *p* < 0.0001, 95% CI (11.4, 16.9)] with greater effects reported during the alcohol, compared to the placebo, condition for both groups. While there was a trend for a group-by-condition interaction on SEAS positive valence/positive arousal (F = 3.0, *p* = 0.09), there were no significant group-by-condition interactions across any subjective response variable. There were no group interactions with time of subjective response (pre- to post-scan subjective response collection). There were no other main effects of group. When covarying total BIS scores, the main effects of group on SEAS positive valence/positive arousal [F = 2.3, *p* = 0.1, 95% CI (−0.8, 5.8)] and SEAS positive valence/negative arousal [F = 1.9, *p* = 0.2, 95% CI (−0.9, 5.0)] were no longer significant. However, significant main effects of group in feeling “drunk” and feeling “effects of alcohol” persisted. All results remained significant when covarying if individuals had a history of AUD or CUD (current or past) and when covarying BAI scores. When exploring effects of medication, we observed individuals on an anticonvulsant reported feeling more drunk [F = 6.3, *p* = 0.02, 95% CI (1, 8.8)] and more effects of alcohol [F = 9.4, *p* = 0.003, 95% CI (2, 9.7)] compared to individuals not on an anticonvulsant. There were no other effects of medication. The SEAS positive valence/negative arousal subscale score was normally distributed and related residuals from models were normally distributed. Feeling drunk was not normally distributed (Shapiro-Wilk *p* < 0.05), however, when testing normality of residuals from models investigating “feeling drunk,” residuals were normally distributed. Other subjective response variables (and their model residuals) were not normally distributed (Shapiro-Wilk *p* < 0.05), however, Q-Q plots suggest data is approaching univariate normality. We log transformed subjective response variables that were not normally distributed, and results reported above remained the same.

### 3.3 Between group differences in placebo response: ROI-ROI approach

**FIGURE 2:**
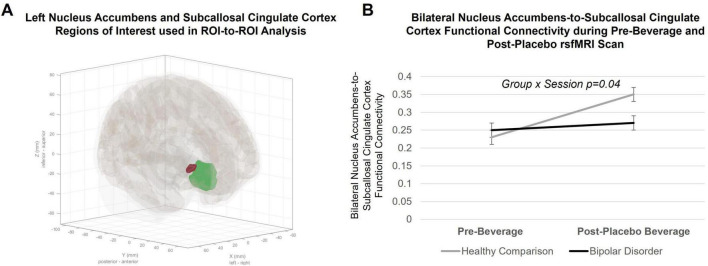
Region of interest (ROI)-to-ROI group-level analysis in the CONN toolbox. **(A)** Anatomical ROIs for the left Nucleus Accumbens (NAc; red ROI) and the Subcallosal Cingulate Cortex (SCC; green ROI) defined in CONN. **(B)** Within-subject changes in bilateral Nucleus Accumbens-to-Subcallosal Cingulate Cortex resting state functional connectivity from pre- to post-placebo beverage consumption. Lines indicate within-subject changes from pre- to post-beverage consumption. Black line indicates young adults with bipolar disorder; gray line indicates healthy comparison young adults. A significant group-by-condition interaction (*p* = 0.04) was observed. *Post hoc* connection-level comparisons indicated a significant increase in NAc-to-SCC functional connectivity during the placebo condition in healthy comparison young adults (F = 13.8, *p* = 0.0006), but not changes in functional connectivity between ROIs in bipolar disorder (F = 0.06, *p* = 0.8).

There was a significant group-by-condition interaction [F = 4.2, *p* = 0.04, 95% CI (0.003, 0.2)] when investigating functional connectivity between the NAc and SCC (see Figure 2). When stratifying by group, the healthy comparison group showed an increase in NAc functional connectivity with SCC during the placebo, compared to the pre-beverage, rsfMRI scan [main effect of condition: F = 13.8, *p* = 0.0006, 95% CI (0.05, 0.2)], while young adults with bipolar disorder did not show a change in NAc-to-SCC functional connectivity [F = 0.06, *p* = 0.8, 95% CI (−0.07, 0.1)]. There were no significant interactions with hemisphere. Functional connectivity between the NAc and vmPFC ROI did not differ by group or show a group-by-condition interaction. Results remained significant in sensitivity analyses. There were no effects of medication. Data and residuals from models were normally distributed.

### 3.4 Between group differences in placebo response (placebo minus pre-beverage rsfMRI): NAc seed region

**FIGURE 3:**
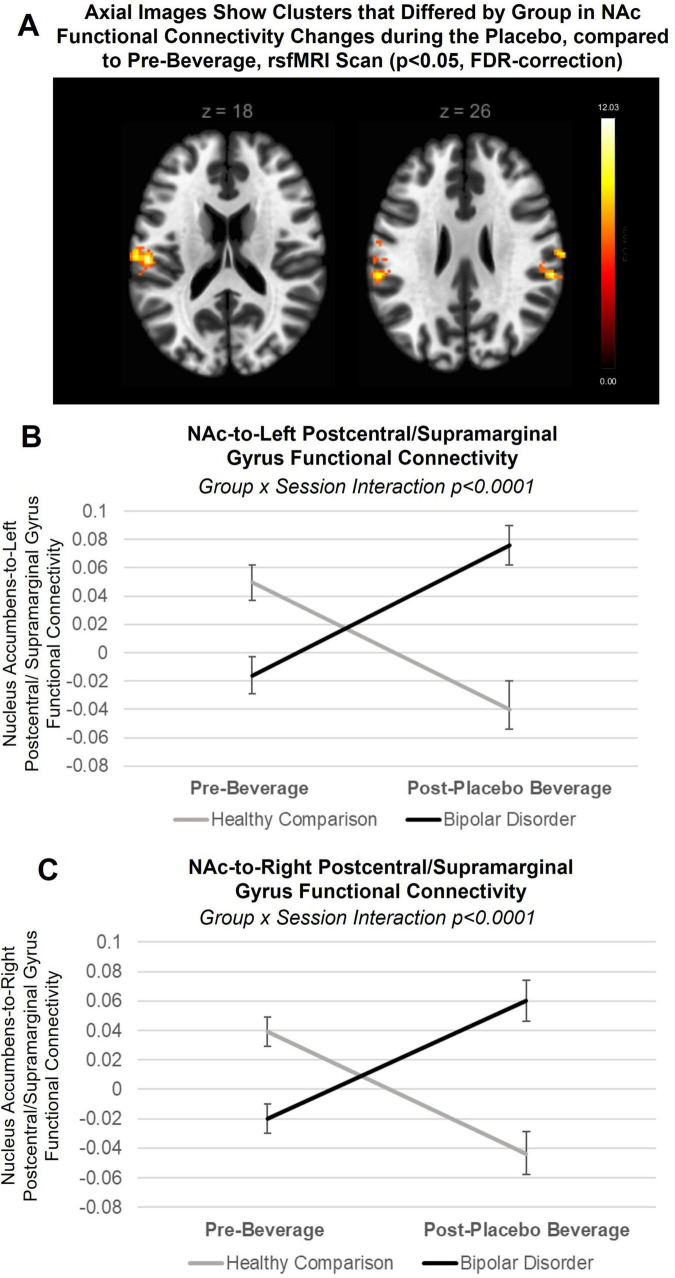
Between group differences in placebo response: nucleus accumbens (NAc) seed-to-voxel approach. **(A)** Axial slices showing clusters that differed by group in NAc functional connectivity changes during the placebo condition, compared to pre-beverage rsfMRI scan (p_FDR_ < 0.05). *Post hoc* analysis revealed group-by-condition (placebo, pre-beverage rsfMRI) interactions (but not interaction with NAc hemisphere) in functional connectivity with both the **(B)** left postcentral/supramarginal gyrus (F = 24.7, *p* < 0.0001) and **(C)** right postcentral/supramarginal gyrus clusters (F = 21.9, *p* < 0.0001). When stratifying by group to interpret the group-by-condition interactions, young adults with bipolar disorder showed a significant increase in NAc functional connectivity during the placebo, compared to pre-beverage rsfMRI scan, with the left postcentral/supramarginal gyrus (β = 0.09, *p* = 0.0004) and the right postcentral/supramarginal gyrus (β = 0.08, *p* = 0.001), while healthy comparison young adults showed a decrease in NAc-to-left postcentral/supramarginal gyrus (β = −0.08, *p* = 0.003) as well as the right postcentral/supramarginal gyrus functional connectivity (β = −0.08, *p* = 0.004). Lines indicate within-subject changes from pre- to post-beverage consumption. Black line indicates young adults with bipolar disorder; gray line indicates healthy comparison young adults.

There was a significant group difference in change in functional connectivity of the NAC during the placebo, compared to pre-beverage rsfMRI scan, with a cluster within the left postcentral gyrus extending into the central and parietal operculum cortex and supramarginal gyrus (NAc-to-left postcentral/SMG cluster: x = −58 mm, y = −30 mm, z = +24 mm; 323 voxels; p-FDR = 0.000009, see Figure 3A) and with a cluster in the right postcentral gyrus extending into the parietal operculum cortex and supramarginal gyrus (NAc-to-right postcentral/SMG cluster: x = +66 mm, y = −16 mm, z = +24 mm; 117 voxels; p-FDR = 0.02). When modeling extracted data, there were no interactions with NAc hemisphere. As seen in Figures 3B, C, when stratifying by group to interpret the group-by-condition interaction on NAc-to-left postcentral gyrus/SMG [F = 24.7, *p* < 0.0001, 95% CI (0.1, 0.25)] and NAc-to-right postcentral gyrus/SMG [F = 21.9, *p* < 0.0001, 95% CI (0.09, 0.23)] functional connectivity, young adults with bipolar disorder showed a significant increase in NAc functional connectivity during the placebo, compared to pre-beverage rsfMRI scan, with the left postcentral/SMG [β = 0.09, *p* = 0.0004, 95% CI (0.04, 0.14)] and the right postcentral/SMG [β = 0.08, *p* = 0.001, 95% CI (0.03, 0.12)], while healthy comparison young adults showed a decrease in NAc-to-left postcentral/SMG [β = −0.08, *p* = 0.003, 95% CI (−0.03, −0.14)] as well as the right postcentral/SMG functional connectivity [β = −0.08, *p* = 0.004, 95% CI (−0.03, −0.14)]. There were no effects of medication. Data and residuals from models were normally distributed.

### 3.5 Placebo-related changes in functional connectivity relations with subjective response to placebo

**FIGURE 4:**
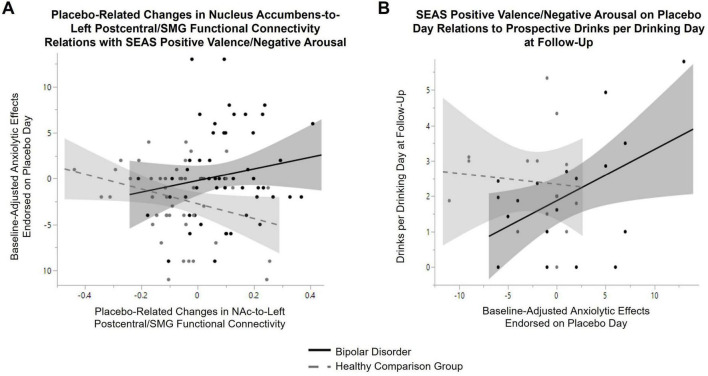
Relations between subjective response to placebo, placebo-related changes in Nucleus Accumbens (NAc) functional connectivity and prospective alcohol use in bipolar disorder. **(A)** There was a significant group-by-placebo-adjusted change in functional connectivity between NAc-to-left postcentral/supramarginal gyrus (F = 4.7, *p* = 0.03) on SEAS positive valence/negative arousal. When stratifying by group, a positive relation between placebo-adjusted NAc-to-postcentral/supramarginal gyrus functional connectivity and anxiolytic effects endorsed during the placebo condition was observed in bipolar disorder (β = 11.8, *p* = 0.02, black line), but not in the healthy comparison group (β = −4.7, *p* = 0.3, gray dashed line). **(B)** A group-by-baseline positive valence/negative arousal score on the SEAS during placebo condition interaction on average drinks per drinking day at follow-up (controlling for baseline drinks per drinking day and time between baseline and follow-up) was observed (F = 5.9, *p* = 0.02). When stratifying by group, a positive relation between anxiolytic effects endorsed during the placebo condition and prospective alcohol use was observed in bipolar disorder (β = 0.2, *p* = 0.01, black line), while there was no significant relation in the healthy comparison group (β = −0.04, *p* = 0.6, gray dashed line).

There was a significant group-by-placebo-adjusted change in functional connectivity between NAc-to-left postcentral/SMG [F = 4.7, *p* = 0.03, 95% CI (1.2, 26.5)] on SEAS positive valence/negative arousal (see Figure 4A). When stratifying by group, a positive relation between placebo-adjusted NAc-to-postcentral/SMG functional connectivity and SEAS positive valence/negative arousal score reported during the placebo session was observed in bipolar disorder [β = 11.8, *p* = 0.02, 95% CI (1.6, 22.0)] but not in the healthy comparison group [β = −4.7, *p* = 0.3, 95% CI (−12.9, 3.5)]. A main effect of placebo-adjusted functional connectivity change in NAc-to-left postcentral/SMG was also observed on SEAS positive valence/positive arousal [F = 6.2, *p* = 0.01, 95% CI (−22.2, −2.5)], with a decrease in functional connectivity associated with greater stimulating effects reported in both groups. Functional connectivity changes between NAc-to-right postcentral/SMG did not relate to subjective response reported during the placebo condition.

### 3.6 Exploratory analysis on prospective alcohol use/problems

Groups did not differ in time between baseline and follow-up (on average 2.1 years in bipolar disorder and 2.3 years in healthy comparison young adults, *t*-test: *p* = 0.4). There was a main effect of time on symptoms of AUD (F = 5.9, *p* = 0.02), with greater symptoms of AUD reported at follow-up across all participants. There was not a significant group-by-time interaction (F = 2.3, *p* = 0.14). Three participants with bipolar disorder converted to meeting threshold for AUD for the first time over the follow-up period (two developed mild and one developed a moderate AUD). No healthy comparison young adult converted to meeting threshold for onset of AUD over the follow-up period. There was no main effect of time (or group-by-time interactions) on prospective alcohol use (measured with the TLFB at baseline and follow-up assessment). A group-by-baseline positive valence/negative arousal score on the SEAS during placebo condition interaction on average drinks per drinking day at follow-up (F = 5.9, *p* = 0.02) and maximum number of drinks per drinking day (F = 4.1, *p* = 0.05) was observed. As seen in Figure 4B, when stratifying by group, a positive relation between SEAS positive valence/negative arousal reported during the placebo session and prospective average drinks per drinking day (β = 0.2, *p* = 0.01) was observed in bipolar disorder. A similar relation in bipolar disorder was observed for maximum drinks per drinking day (β = 0.26, *p* = 0.03). These relations with SEAS positive valence/negative arousal was not observed in the healthy comparison group (average drinks: β = −0.04, *p* = 0.6; maximum drinks: β = −0.11, *p* = 0.5). A positive relation between SEAS positive valence/negative arousal and number of heavy episodic drinking days at follow-up was also observed across both groups (main effect: β = 0.14, *p* = 0.03). A significant positive relation between placebo-related changes in left NAc-to-left postcentral/SMG functional connectivity with AUD symptoms meeting threshold on the SCID at follow-up (β = 5.7, *p* = 0.008) was also observed in bipolar disorder. No other significant relations between subjective response reported during the placebo condition or placebo-related changes in NAc functional connectivity with alcohol-related outcomes in bipolar disorder were observed.

## 4 Discussion

Our results suggest that youth with bipolar disorder show differences in placebo response compared to healthy comparison young adults, with stronger alcohol expectancies (greater stimulating, anxiolytic, and feeling “drunk/effects of alcohol”) during the placebo beverage condition in addition to the alcohol beverage condition observed in young adults with bipolar disorder. Youth with bipolar disorder also showed differences, compared to healthy young adults, in NAc functional connectivity changes during the placebo condition rsfMRI scan compared to a pre-beverage rsfMRI. Specifically, while healthy comparison young adults showed increases in NAc-to-SCC functional connectivity, young adults with bipolar disorder did not. Young adults with bipolar disorder demonstrated increases in NAc-to-postcentral/SMG functional connectivity during the placebo condition, compared to pre-beverage rsfMRI session, which was not observed in healthy comparison young adults. Findings suggest distinct neural correlates of placebo response in bipolar disorder, compared to healthy comparison young adults. Increased NAc-to-postcentral/SMG functional connectivity during the placebo condition related to greater anxiolytic effects reported during placebo in young adults with bipolar disorder (but not healthy comparison young adults). Differences in expectations of anxiolytic effects after consuming alcohol has previously been reported to relate to alcohol use in bipolar disorder (Lippard et al., 2023). While preliminary, longitudinal data reported here suggest a positive association between anxiolytic alcohol expectancies (observed during the placebo condition) and quantity of alcohol use at follow-up in bipolar disorder. A positive association between changes in left NAc-to-postcentral/SMG functional connectivity during the placebo condition and prospective symptoms of AUD was also observed in young adults with bipolar disorder. These findings extend prior work in healthy adults suggesting neural responses to a placebo beverage condition increases risk for future alcohol problems (McKenna et al., 2022). While longitudinal data should be interpreted with caution, preliminary longitudinal results serve as proof of concept for future studies of placebo response in bipolar disorder. Findings highlight the young adult period as a critical time in risk for, and development of, alcohol problems in bipolar disorder and that variability in placebo response may relate to prospective alcohol use.

The postcentral and supramarginal gyrus are key nodes in the sensorimotor network, exhibit high subcortical inputs (for bottom-up processing of sensory information), and are involved in interoceptive and exteroceptive awareness (Salvato et al., 2020; Stern et al., 2017; Wang et al., 2019). Variability in functional connectivity of regions within the sensorimotor network (Bi et al., 2022) and differences in NAc function during rewards anticipation are reported in bipolar disorder (Caseras et al., 2013; Nusslock et al., 2012). Variability in function within the sensorimotor network is also suggested to relate to reward and loss anticipation in bipolar disorder (Manelis et al., 2016). Additionally, a role of the postcentral and supramarginal gyrus is also suggested in AUD (Florence et al., 2022; Gowin et al., 2013; Syan et al., 2023). Findings converge to suggest a role of the sensorimotor network—and subcortical connections—in alcohol expectancies (and possibly rewarding properties of alcohol that may underlie alcohol seeking behavior) in bipolar disorder and could serve as targets for intervention. Interestingly, variability in function within the sensorimotor network, i.e., supramarginal gyrus, is also suggested to relate to placebo analgesia (Kong et al., 2006; Nemoto et al., 2007; Zunhammer et al., 2021). While speculative, future research might investigate if placebo response relates to pain relief—in addition to reward anticipation—in bipolar disorder and whether it could underlie greater coping drinking motives previously reported (Tretyak et al., 2022). As variability in NAc-to-postcentral gyrus functional connectivity is observed in youth with family history of AUD (Cservenka et al., 2014), future research investigating familial and psychosocial factors that contribute to alcohol expectancy development and maintenance in bipolar disorder is needed.

Variability in alcohol expectancies could relate to familial risk factors. Bipolar disorder and AUD often co-aggregate in studies on familial risk for bipolar disorder (Wilens et al., 2014). Alcohol related norms and alcohol expectancies emerge during childhood and are influenced by family and sociocultural factors (Schor, 1996; Skylstad et al., 2022; Smit et al., 2018). Familial factors are also supported as contributing to variability in alcohol cue reactivity even in alcohol naïve adolescents (Nguyen-Louie et al., 2018). Additionally, early life stress is suggested to relate to variability in alcohol expectancies and interact with familial risk to contribute to subjective response to alcohol and alcohol use (Kirsch et al., 2021; Kosted et al., 2023). Interactions between alcohol and stress that affect behavioral and neural plasticity (Brancato et al., 2023) could also impact alcohol-related outcomes, including alcohol expectancies, and early interventions focused on family support may foster resiliency and improved outcomes (Castelli et al., 2020). Collectively these results point to psychosocial factors (for example, familial risk for alcohol use problems, environmental stress, parental attitudes toward alcohol, alcohol availability, and parental support and monitoring) that may interact with genetic vulnerability to contribute to alcohol use in bipolar disorder. While these psychosocial factors may generalize across diagnostic boundaries, they may also be distinct from those underlying risk in healthy young adults—as previously discussed (Tretyak et al., 2022; Tretyak et al., 2021).

Several limitations of this study must be noted to frame interpretation. We cannot rule out type I errors; future study with larger sample size is needed to confirm and extend these findings. Many confounding factors, including medication effects, mood state variability, or comorbid conditions could also influence alcohol response. While youth with bipolar disorder were euthymic at the time of study they varied in subthreshold mood symptoms. When covarying subthreshold anxiety symptoms, results remain the same. When covarying impulsivity scores, the main effect of group on anxiolytic and stimulating effects of alcohol was no longer significant. It remains unclear whether impulsivity is a confounding variable, a mechanistic factor, or a potential mediator/moderator. Future research on the role(s) of impulsivity on subjective response to alcohol (and placebo/alcohol expectancies) is needed. There is existing literature regarding subjective responses to drugs, e.g., amphetamine, suggesting relations with personality traits including impulsivity (Kirkpatrick et al., 2013; Weafer and de Wit, 2013). However, distinct relations between subjective response to alcohol, compared to other drugs, are also reported (Wardle et al., 2015). We cannot say if findings would generalize to other substances and might contribute to other substance use comorbidities in bipolar disorder. While we observed that anticonvulsant use was associated with young adults with bipolar disorder reporting feeling more drunk/effects of alcohol, this study was not powered to investigate the impact of medication on subjective response to alcohol. Effects of medication should be interpreted with caution. Future research investigating effects of medication on development of alcohol misuse over time is needed to inform treatment decision-making in bipolar disorder, especially in individuals who are starting to show signs of alcohol use problems. Medication did not relate to placebo-associated changes in NAc functional connectivity observed in bipolar disorder. This study focused on individuals with bipolar disorder type I. While this decreased heterogeneity in the sample, it does limit our ability to generalize findings to other subtypes.

This manuscript is intended to focus more on placebo response (a measure of alcohol expectancy). BrAC varied within each group across their alcohol condition and could have contributed to differences in subjective response to alcohol; this effect is less of a concern when investigating placebo response. Other methods of alcohol administration (i.e., IV administration) could control variability in BrAC during the alcohol condition but the oral consumption used in this study strengthens the placebo manipulation and increases ecological validity. The fMRI data used in this study was during a pre-beverage session and after individuals had consumed the placebo beverage (not the beverage containing alcohol). The resting state findings reported are therefore not a direct effect of alcohol pharmacokinetics. It is possible differences observed following the placebo beverage consumption (compared to pre-beverage rsfMRI scan) could relate to alcohol cue exposure. This proof-of-concept study highlights the need for future work on alcohol expectancies/placebo response in bipolar disorder and risk for future alcohol problems. Future work should include counter-balanced “Told No Alcohol/Get No Alcohol” (no alcohol expectancy) and “Told Alcohol/Get No Alcohol” (alcohol expectancy) conditions. Both groups had more women than men. Risk for AUD in bipolar disorder is suggested to differ between men and women (Lippard et al., 2017), and gender differences in subjective response to alcohol and alcohol expectancies are reported (Ide et al., 2017; Satre and Knight, 2001; Schuckit et al., 2012; Schultz et al., 2021). Additionally, studies suggest in general males may respond more strongly to placebo effects (Vambheim and Flaten, 2017) although greater placebo response in women to some drugs are reported (Singha et al., 2000). Collectively, these results emphasize a need for research investigating gender differences; we were not powered to investigate gender differences in the current study. Older adults, compared to younger adults, report differences in alcohol expectancies (Satre and Knight, 2001). While the homogeneous age range (21–26 years of age) decreased heterogeneity and focused on a period of risk for alcohol misuse, findings may not generalize to older samples. Increased left SMG activity has been reported following expectancy violation and to correlate with decreased placebo response (Colloca et al., 2019). However, we excluded individuals from these analyses that reported not believing the placebo manipulation and no group difference in estimated number of drinks consumed was noted during the placebo manipulation check. While there was no difference in recent alcohol or cannabis use between groups at enrollment, we cannot rule out groups may have differed in past alcohol or cannabis use which could have contributed to variability in placebo response. We cannot discern the molecular mechanisms that may underly variability in placebo response. The rewarding/activating and sedating effects of alcohol may be mediated by the dopaminergic and GABAergic systems, respectively. Dopamine and GABA dynamics may contribute to variability in alcohol expectancies, suggesting a biological influence on some cognitions underlying alcohol use (Young et al., 2004). While we did not observe group differences in the sedative effects reported following beverage consumption (measured with the SEAS), more research on dopamine and GABA dynamics and interactions with alcohol/substance use that may drive development of expectancies in bipolar disorder is needed. Our group with bipolar disorder had more individuals with a history of AUD as well as CUD. We did not exclude individuals with a mild/moderate AUD or a history of CUD to avoid limiting generalizability. It is possible that this difference contributed to variability in alcohol expectancies. However, when covarying for individuals with a history of AUD or CUD in sensitivity analyses, the results remained significant, suggesting differences in alcohol expectancies may predate the development of AUD or CUD in individuals with bipolar disorder. Additionally, co-use of alcohol and cannabis is suggested to interact to alter subjective response to alcohol and contribute to drinking behavior (Waddell et al., 2023, 2024) and we cannot rule out the possibility that differences in patterns of co-use might have contributed to placebo findings. Findings may not generalize to heavier drinkers and those with AUD. We also cannot rule out effects of medication as most individuals with bipolar disorder were medicated at the time of this study. *Post hoc* analysis of NAc-to-SGM may suffer from sequential testing issues, i.e., variables used to identify the effect being statistically related to the variables tested *post hoc*. We recently reported acute alcohol-related changes (compared to placebo condition) in NAc-to-prefrontal cortex and amygdala-to-prefrontal cortex functional connectivity in bipolar disorder that related to subjective response to alcohol (Kirsch et al., 2023a). We did not observe NAc-to-prefrontal cortex functional connectivity changes that related specifically to placebo response in bipolar disorder in this analysis. As prior work suggests interactions between anticipated effects and subjective response to alcohol on alcohol-related outcomes in healthy adults (Morean et al., 2015; Waddell et al., 2022), future work with the power to investigate interactions between placebo response and acute alcohol effects on alcohol-related outcomes in bipolar disorder is needed. Participants drank in a controlled non-bar setting. Alcohol cue exposure in a bar context is suggested to enhance alcohol expectancies and craving compared to a non-bar context (Chen et al., 2021; Corbin et al., 2015; Kuerbis et al., 2020). Additionally, in this study the participant was the only one drinking (although study personnel were with the participant). While the use of oral alcohol administration improves ecological validity, the controlled setting (where participants were drinking alone in a non-bar setting) differs from real-world social drinking experiences. Variability in subjective response to alcohol is observed in different social contexts [group vs. solitary drinking (Corbin et al., 2021)] and modifying the drinking context and the rewarding value of drinking (Corbin et al., 2008; Sayette et al., 2012) might alter differences in subjective response than those reported here. Increases in sensitivity to alcohol is suggested to relate to maintenance/escalation of AUD (King et al., 2021). Variability in sensitivity to alcohol may emerge over time and relate to AUD onset/maintenance in bipolar disorder. We are not able to determine the temporal associations between placebo response and alcohol use. It is possible that variability in subjective experience of alcohol (at time of alcohol initiation) contributes to variability in alcohol expectancies in bipolar disorder. It is premature to disentangle if findings reported here relate to risk or resiliency for future alcohol problems in bipolar disorder. It is possible young adults with bipolar disorder recruited for this study are a biased “healthier” cohort that may be more resilient to development of AUD. We can only speculate the level of risk for AUD in this sample. If the sample underrepresents individuals at the highest risk for AUD, results could represent an underestimation of the true relationship between placebo response and alcohol use in bipolar disorder. While when looking at preliminary longitudinal data, placebo response related to prospective alcohol use and problems being reported in bipolar disorder, more longitudinal data with larger sample sizes and longer periods of follow-up are needed.

In summary, this analysis suggests that young adults with bipolar disorder show differences in alcohol expectancies (suggested by elevated placebo response), with variability in alcohol expectancies relating to changes in NAc-to-sensorimotor network functional connectivity and future alcohol use. As emerging data suggest alcohol expectancies can be targeted for interventions to improve alcohol-related outcomes (Dunn et al., 2020; Dunn et al., 2022; Lau et al., 2022; Schick et al., 2022), future research in this area is needed. Specifically, longitudinal studies powered to capture the emergence of alcohol problems are necessary to investigate unique alcohol effects that may interact with alcohol expectancies to contribute to risk for future alcohol use problems in bipolar disorder. Ultimately, this line of research might inform interventions and prevention efforts that are more effective in youth with bipolar disorder than existing treatments.

## Data Availability

The raw data supporting the conclusions of this article will be made available by the authors, without undue reservation.

## Footnotes

1
www.nitrc.org/projects/conn
